# Carboxyl of Poly(D,L-lactide-co-glycolide) Nanoparticles of Perfluorooctyl Bromide for Ultrasonic Imaging of Tumor

**DOI:** 10.1155/2018/2957459

**Published:** 2018-02-07

**Authors:** Shengjuan Luo, Jinsong Ding, Peiqi Wang, Zheng Wang, Xiaoqian Ma, Cejun Yang, Qi Liang, Pengfei Rong, Wei Wang

**Affiliations:** ^1^Department of Ultrasound, The Third Xiangya Hospital, Central South University, Changsha 410013, China; ^2^School of Pharmaceutical Sciences, Central South University, Changsha 410013, China; ^3^Department of Pharmacy, Cancer Hospital of Henan Province, Zhengzhou 450008, China; ^4^Department of Hepatobiliary Surgery, The Third Xiangya Hospital, Central South University, Changsha 410013, China; ^5^Department of Radiology, The Third Xiangya Hospital, Central South University, Changsha 410013, China

## Abstract

Perfluorooctyl bromide (PFOB) enclosed nanoparticles (NPs) as ultrasonic contrasts have shown promising results in the recent years. However, NPs display poor contrast enhancement in vivo. In this work, we used the copolymers poly(lactide-co-glycolide) carboxylic acid (PLGA-COOH) and poly(lactide-co- glycolide) poly(ethylene glycol) carboxylic acid (PLGA-PEG-COOH) as a shell to encapsulate PFOB to prepare a nanoultrasonic contrast agent. The NPs were small and uniform (210.6 ± 2.9 nm with a polydispersity index of 0.129 ± 0.016) with a complete shell nuclear structure under the transmission electron microscopy (TEM). In vitro, when concentration of NPs was ≥10 mg/ml and clinical diagnostic frequency was ≥9 MHz, NPs produced intensive enhancement of ultrasonic gray-scale signals. NPs could produce stable and obvious gray enhancement with high mechanical index (MI) (MI > 0.6). In vivo, the NPs offered good ultrasound enhancement in tumor after more than 24 h and optical imaging also indicated that NPs were mainly located at tumor site. Subsequent analysis confirmed that large accumulation of fluorescence was observed in the frozen section of the tumor tissue. All these results caused the conclusion that NPs encapsulated PFOB has achieved tumor-selective imaging in vivo.

## 1. Introduction

In recent years, ultrasonic molecular imaging has become a promising method for cancer diagnostics because it can image an intact living body at cellular and subcellular level with high spatial and temporal resolution, low cost, portability, and lack of ionizing irradiation [1]. Ultrasound contrast agents (UCAs) are necessary for ultrasound molecular imaging, and they can improve the accuracy and sensitivity of ultrasound diagnosis [2]. Currently, the contrast agents in the market consist of gas-encapsulated phospholipids or albumin (1 to 8 microns) [3]. However, most tumors have porous vasculature with fenestrations between 380 and 780 nm [4]. The micro-sized UCAs are often limited by their lack of efficient penetration. To overcome this limitation, decreasing the UCA size to nanometer range would make UCA more likely to penetrate into tumor tissue.

Nanobubbles were used in most studies related to nanoscale UCAs because they produced good sound reflection [5–8]. However, in vitro nanobubbles would quickly break or fuse without pressure damage [8]; in vivo, nanobubbles could be removed by the reticuloendothelial system (RES) [9]; ultrasound also had some damage to them [10]. Therefore, the number of nanobubbles that went through the circulation to the tumor and inflammation was small, which would limit their ability of target diagnosis and treatment. Nanoparticles (NPs) with liquid perfluorocarbons as the core were more resistant to pressure changes and mechanical stresses. When bounding and gathering at a specific target, nanoparticles of liquid perfluorocarbons could produce strong signals but negligible signals in circulation [11, 12]. Perfluorooctyl bromide (PFOB) is of low toxicity and stability. It is the most suitable liquid perfluorocarbon to be used in vivo [13]. Inferior vena cava of the nude mouse presented significant gray enhancement for a few seconds after being injected with nanoparticles of PLGA enclosed PFOB [14]. Because of the hydrophobic properties of PLGA, nanoparticles were quickly cleared by RES in vivo. At present, the most effective and widely used method is to use nonionized polymer polyethylene glycol (PEG) to modify the PLGA [9, 15]. DSPE-PEG was used to modify PLGA and it was found that the dosage of DSPE-PEG affected the core-shell structure of NPs. When the dosage of DSPE-PEG is >2.64 mg (100 mg PLGA), NPs could not maintain a complete core-shell structure, but when the dosage of DSPE-PEG is ≤2.64 mg, only 6% of DSPE-PEG in the shell, NPs with a complete core-shell structure could not prevent themselves from being cleared by RES and could not gather in the tumor for imaging [13]. In order to maintain the stability of the core-shell structured nanoparticles and have a good ultrasonic contrast enhancement, it is necessary to use the compatible material packaged PFOB to carry enough PEG to avoid RES.

Here, we would use hybrid membrane materials of PLGA-PEG-COOH and PLGA-COOH packaged PFOB to prepare nanoscale UCAs, which all had complete core-shell structure with a good gray enhancement in the aqueous solution in vitro. In in vivo experiments, the enhancement of ultrasound gray scales at the tumor site was analyzed after tail intravenous injection. Finally, tumor-selective imaging of NPs was assessed by both small animal optical imaging and fluorescence microscope.

## 2. Materials and Methods

### 2.1. Materials

Poly(D,L-lactide-co-glycolide) (50/50) with terminal carboxylate groups (PLGA-COOH, Mw = 17000) was purchased from Jinan Daigang Biotech Co., Ltd. (Jinan, Shandong, China). NH_2_-PEG-COOH (Mw = 3400) was purchased from Beijing Kaizheng Biotech Co., Ltd. (Beijing, China). Polyvinyl alcohol (Mw 13000–23000, 98% hydrolyzed), 4′-6-diamidino-2-phenylindole (DAPI), and coumarin-6 were provided by Sigma-Aldrich (St. Louis, MO). Perfluorooctyl bromide (PFOB) was purchased from Aladdin Industrial Corporation (Shanghai, China). HepG2 cells (human liver hepatocellular carcinoma cell line) were purchased from the Cell Institute of the Chinese Academy of Sciences (Shanghai, China). All other reagents were of analytical pure grade and were purchased from Sinopharm Chemical Reagent Co., Ltd. (Shanghai, China).

### 2.2. Synthesis of PLGA-PEG-COOH Copolymer

**Figure 1:**
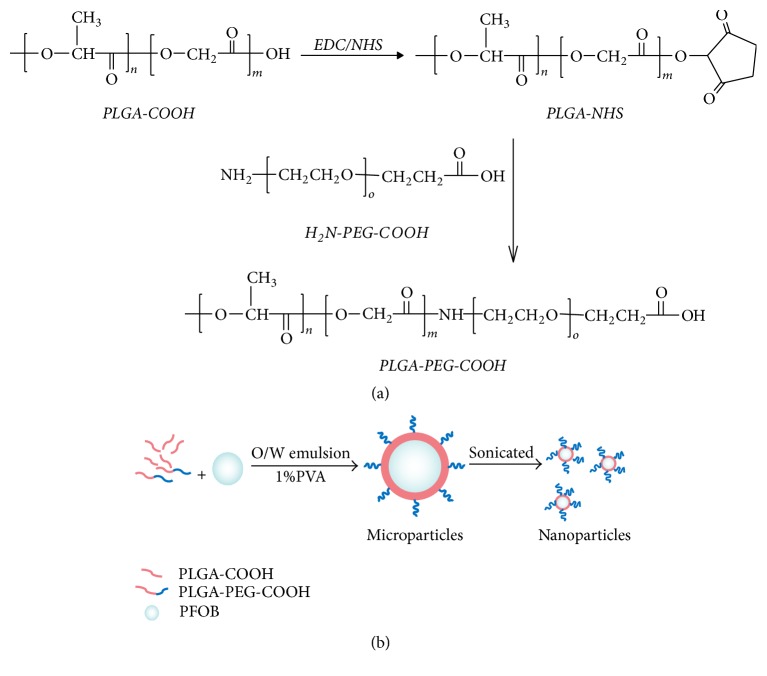
Synthesized PEG-PLGA-COOH copolymer in CDCl_3_ (a). Schematic representation of the preparation process of nanoparticles for ultrasonic imaging (b).

Carboxylate-functionalized copolymer PLGA-PEG was synthesized by conjugating COOH-PEG-NH_2_ to PLGA-COOH according to a carbodiimide–N- hydroxysuccinimide (EDC/NHS)-mediated chemistry [16] (Figure 1(a)). The copolymer was dissolved in CDCl_3_ and characterized by ^1^H NMR at 400 Hz (AVANCE III 400 M, Bruker, Billerica, MA) to determine the modification ratio of PEG on PLGA.

### 2.3. Preparation of Nanoparticles/Microparticles Encapsulated PFOB

PFOB was encapsulated in nanoparticles by modifying the emulsion solvent evaporation method [17] (Figure 1(b)). Briefly, 50 mg PLGA blended membrane materials were dissolved in 2 ml methylene chloride along with 30 *μ*l PFOB. The organic solution placed in a thermostatic bath was maintained at 20°C to ensure full miscibility of the PFOB. This was then emulsified into 10 ml 1.0% polyvinyl alcohol (PVA; w/v) aqueous solution to form a preemulsion. The preemulsion was sonicated at 300 W for 2 min over ice. Organic solvents were then evaporated for 3 h in a thermostatic bath (30°C) to remove the methylene chloride. To acquire microparticles encapsulated PFOB (MPs) as contrast, the organic solvents were evaporated immediately after the preemulsion's formation. NPs/MPs labeled coumarin-6/DiR were prepared by adding 50 *μ*l coumarin-6/DiR to the organic solution prior to emulsification to label the polymer phase.

### 2.4. Measurement of NPs Characteristics

Particle diameter, size distribution, and zeta potential of the NPs/MPs were measured by a dynamic light-scattering system (DLS; Zetasizer Nano-ZS; Malvern Instruments, Worcestershire, England) at 25°C. The structure of the NPs was examined by transmission electron microscopy (TEM; Tecnai™ G2 Spirit TWIN, Netherlands). The morphology of microparticles was observed by optical microscope (Olympus, Japan).

### 2.5. Encapsulation Efficiency (EE)

A weighed amount of NPs was dissolved into methanol following 5 min ultrasonic treatment and then centrifuged for 10 min. PFOB concentration of supernatant liquor was measured by gas chromatograph (GC) at 300°C, using flame ionization detector (FID). Percentages of encapsulation efficiency (% EE) were calculated based on the following equations:

(1) $$\begin{array}{c} \%\text{ EE}=\frac{\text{drug entrapped in NPs}}{\text{initial amount of drug added}}\times 100. \end{array}$$

### 2.6. In Vitro Echogenicity of Contrast Agents Study

Each NP/MP suspension sample was filled in an Eppendorf tube. Ultrasound images were obtained in a nonlinear mode with a commercial ultrasound imaging system (L 74M probe, HI VISION Ascendus, Hitachi, Japan). All images were acquired using the same instrument parameters: frame rate (FR) 26, brightness (BG) 20, and dynamic range (DR) 65 db.

### 2.7. Tumor-Bearing Mouse Model

Nude male BALB/c mice (age of 5 weeks) were obtained from Hunan Slake Jingda Experimental Animal Co., Ltd. (Changsha, China). Approximately 1.0 × 10^7^ HepG2 cells were inoculated subcutaneously into the right hind legs of the mice. All in vivo experiments began when the diameter of tumors reached 0.8–1.2 cm. The laboratory animal management committee and ethics committee at the Third Xiangya Hospital of Central South University approved all animal experiments.

### 2.8. In Vivo Echogenicity of Contrast Agents

Tumor-bearing nude mice were divided into two groups at random: the control group (three mice injected with MPs) and the experimental group (six mice injected with NPs). No animal deaths occurred in the experimental process.

Mice were anesthetized with 1% pentobarbital sodium by abdominal injection (1 mg/100 g); 0.3 ml samples were injected into the body via the tail vein. Images were collected before and after injection: 0.5 h, 2 h, 12 h, 24 h, and 48 h, using a L74M transducer with 10 MHz and MI of 1.0. No instrument parameters changed during this experiment. All data and images were stored for offline analysis. Because of individual differences and tumor heterogeneity, gray-scale images were quite different. We defined the quantitative gray scale as follows.

(2) $$\begin{array}{c} \text{The increased rates}=\frac{{RGV}_{x}-{RGV}_{0}}{{RGV}_{0}}. \end{array}$$

RGV_0_ referred to image gray-scale intensity before the injection, and RGV_*x*_ referred to image gray-scale intensity after the injection [6].

### 2.9. Optical Imaging

IVIS Lumina II (Caliper, Alameda, CA, USA) was used for in vivo optical imaging. NPs and MPs containing DiR were injected into 2 groups of mice (3 mice per group). Then mice were put into an opaque black box. The excitation filter of DiR was 745 nm and emission filter of it was 810–875 nm. Images were obtained by the CCD camera at 0 h, 0.5 h, 2 h, 12 h, 24 h, and 48 h. All the data were analyzed by Living Image® Software 4.0 (Caliper, Alameda, CA, USA).

### 2.10. Histological Analysis

NPs or MPs labeled coumarin-6 was injected into two groups of mice (3 mice per group). After being injected for 24 h, tumor-bearing mice were killed, and collected tumors were sectioned into 5 *μ*m slices. Frozen sections were stained with DAPI for labeling the nuclei of tumor cells. Images were obtained by using a fluorescence microscope (Olympus). DAPI and coumarin-6 were excited at 340 and 466 nm, respectively, and the emission was recorded at 488 and 504 nm, respectively.

### 2.11. Statistical Methods

All experiments were conducted in triplicate. All data were expressed as the mean ± SD. Statistical analyses were performed using one-way analysis of variance (ANOVA) and *t* test.

## 3. Results

### 3.1. Synthesis and Characterization of PLGA-PEG

**Figure 2:**
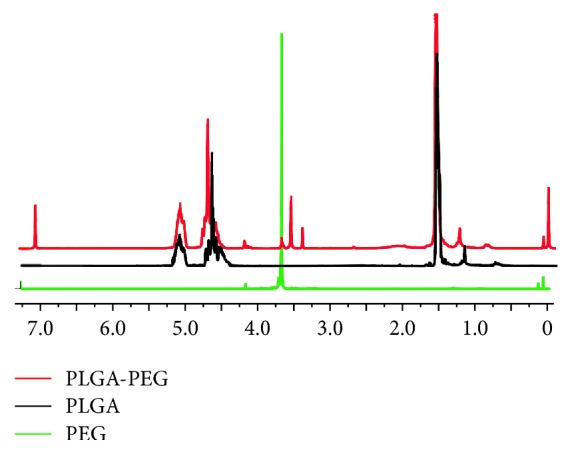
^1^H-NMR spectra of PLGA, PEG, and PLGA-PEG.

The chemical composition of the synthesized product was confirmed by ^1^H-NMR (Figure 2). The characteristic peaks at 1.5, 4.8, and 5.2 ppm belonged to the methyl (d, -CH_3_), methane (m, -CH_2_), and methine (m, -CH) proton of the PLGA segment, respectively. The peak at 3.7 ppm was associated with the methene (s, -CH_2_) proton of the PEG chain. By using the relative molecular weights and the integration of characteristic peaks at 5.20 and 3.7 ppm, the conjugation efficiency of NH_2_-PEG-COOH to PLGA-COOH was estimated to be 12.5%.

### 3.2. Characterization of NPs

**Figure 3:**
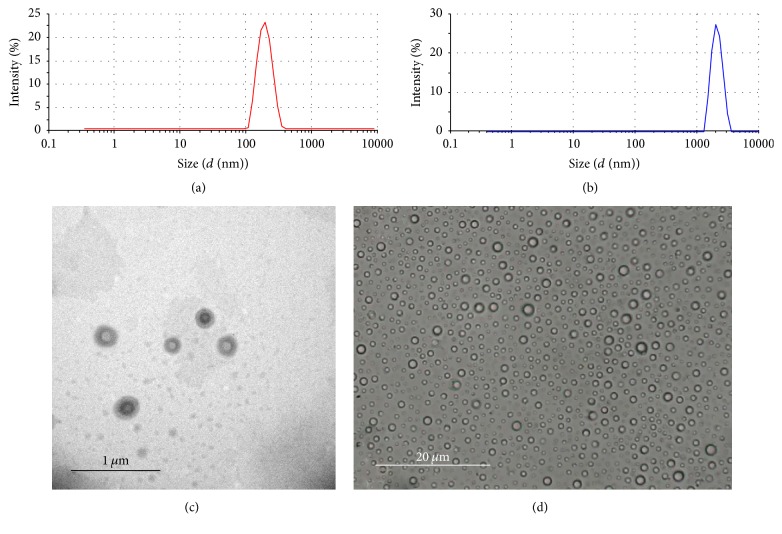
Particle size of the NPs and MPs. The size distribution was measured using dynamic light scattering in the NPs (a) and MPs (b). The morphologies of the NPs (c) and MPs (d) were determined by transmission electron micrographs and optical microscope, respectively.

The size distribution and zeta-potential of the NPs and MPs were assessed. The mean diameter of the NPs was 212.7 ± 2.76 nm (Figure 3(a)) with a polydispersity distribution (PDI) of 0.16 ± 0.03. In contrast, the mean diameter of the MPs was 2480.4 ± 380.4 nm (Figure 3(b)) with a PDI of 0.32 ± 0.037. The zeta-potential of the NPs was −32.7 ± 1.02 mV. MPs had a zeta-potential of −12.5 ± 2.03 mV. Under TEM, the NPs (Figure 3(c)) were spherical and had an intact and homogeneous shell. Because of the different electronic densities, the shell seemed darker than the gray core. MPs observed by optical microscopy were all core-shelled (Figure 3(d)). Encapsulation efficiency of the nanoparticles was 80.43 ± 0.96%, and the concentration of PFOB in NPs solution was 10.09 mg/ml.

### 3.3. In Vitro Ultrasound Imaging

**Figure 4:**
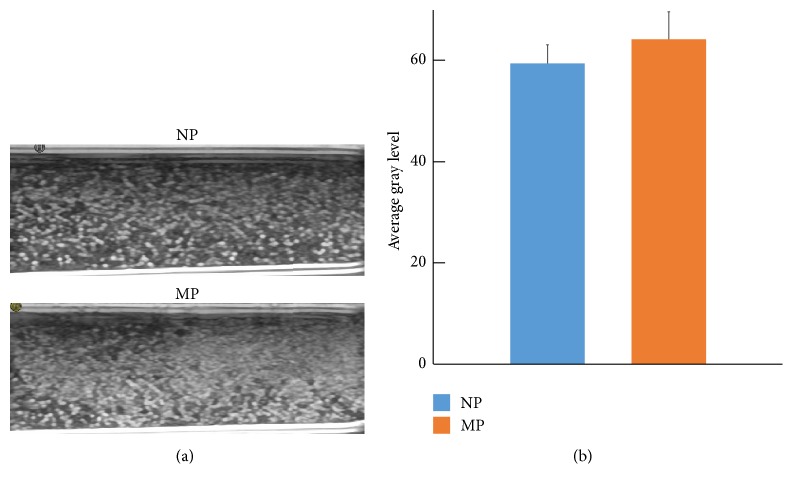
(a) Ultrasound imaging of NPs and MPs at 10 MHz in vitro. (b) Gray-scale ultrasonic intensity of NPs and MPs. NPs presented similar gray-scale intensity to MPs (*P* = 0.361).

To compare the ultrasonic reflectance ability of NPs with that of MPs, in vitro ultrasound imaging was acquired by using diagnostic high-frequency ultrasound (10 MHz) (Figure 4(a)). The results showed that, with the same PFOB concentration of NPs and MPs (10 mg/ml), the ultrasonic signals of NPs were weaker, but there was no statistical difference between the two groups (Figure 4(b)).

**Figure 5:**
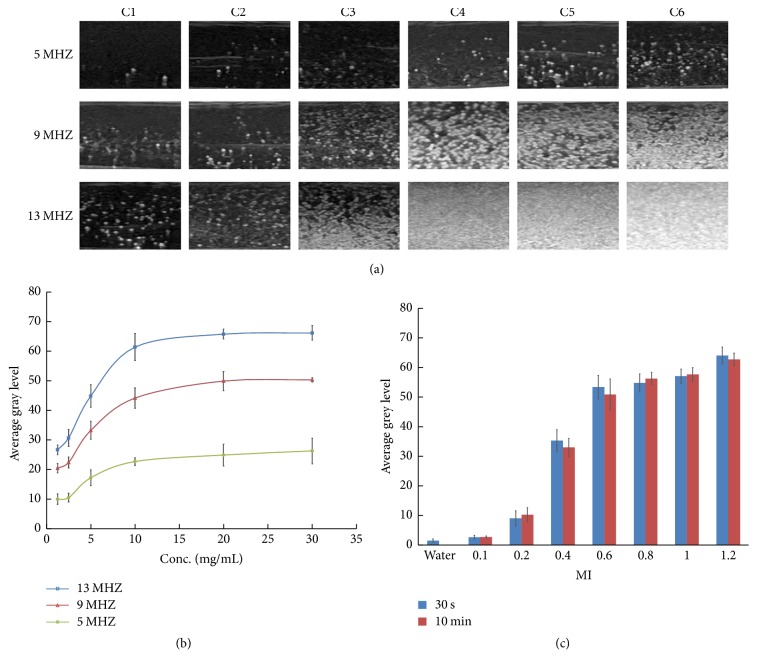
(a) Ultrasound images obtained in vitro in nonlinear mode at different concentration at 5, 9, and 13 MHz. C1: 1.25 mg/ml, C2: 2.5 mg/ml, C3: 5 mg/ml, C4: 10 mg/ml, C5: 20 mg/ml, and C6: 30 mg/ml. (b) Effect of nanoparticles concentration on echographic image brightness with different probe frequency at 5, 9, and 13 MHz. (c) Effect of mechanical index on echographic image brightness.

We also studied the influence of concentration and probe frequency on ultrasonic signals produced by NPs. Enriched NPs were diluted with degassing deionized water (C6: 30 mg/ml; C5: 20 mg/ml; C4: 10 mg/ml; C3: 5 mg/ml; C2: 2.5 mg/ml; and C1: 1.25 mg/ml).  Figure 5(a) showed ultrasonic reflection images of the NPs of six different concentrations at three different frequencies (5, 9, and 13 MHz). When the concentration and the frequency were the lowest, the echo reflection of the solution was the lowest (C1−5 MHz). The signal was weak at 5 MHz (C6-5 MHz) even if the concentration was high (>10 mg/ml). The signal obviously enhanced at 9 MHz or 13 MHz especially when the concentration of NPs was ≥10 mg/ml. The gray-scale concentration relationship was shown in Figure 5(b). The former part of steeper curves of 13 MHz and 9 MHz revealed a linear-like relationship when concentration was from 1.25 to 10 mg/ml, and the latter part of the curves became even when concentration was ≥10 mg/ml. The curve at 5 MHz as a whole was comparatively flat.

We also separately studied the influence of mechanical index (MI) on NPs echo reflection. We selected a 10 mg/ml sample as the research object.  Figure 5(c) showed that the image gray levels became higher with increasing MI. When the MI was 0.1, the echo reflection of the sample was close to the echo reflection of degassed deionized water. The contrast enhancement did not become weak at MI > 0.6 even if the contrast agent was exposed to ultrasound for 10 minutes.

### 3.4. Ultrasonic Imaging of Tumor-Burdened Mice

**Figure 6:**
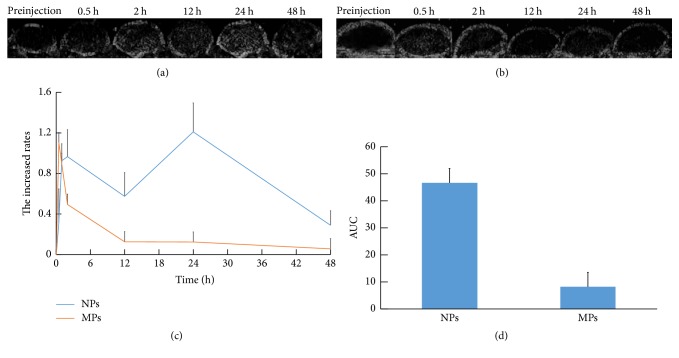
In vivo ultrasonic imaging of tumor-burdened mice. Representative subcutaneous tumor images before and after the injection of nanoparticles (NPs) (a) compared with MPs (b) at various time points (preinjection, 0.5 h, 2 h, 12 h, 24 h, and 48 h). Corresponding time-intensity curves of tumor enhancement after injection of the contrast agent (c). (d) AUC analysis with data was extracted from figure (c) (*P* = 0.001).

Tissue harmonic imaging- (THI-) mode imaging was carried out on two groups of tumor-bearing mice. The tumor images of the contrast enhancement were provided by NPs (Figure 6(a)) and MPs (Figure 6(b)). Tumor images showed obvious enhancement in the NP and MP groups at different time. Intensity-time diagrams of the tumors were illustrated in Figure 6(c). In the NP group, the increased rates (TIR) slowly rose after injection. The TIR was 30% 0.5 h after injection and two hours later, the TIR was 97%. The ultrasound enhancement effect produced by NPs was clearly distinguishable. After then, enhanced intensity strengthened continuously. 24 h after injection, contrast enhancement of NPs at the tumor site was still evident (TIR = 121%). This trend is different from that in MP group which had a rapid wash-in and washout. Time-intensity curve showed that ultrasound gray enhanced and reached the peak (TIR = 110%) at 0.5 h after being injected with MPs, and then it weakened quickly. In order to further compare the contrast enhancements in NP and MP groups, the area under the curve (AUC) plotted after injection from 0 to 48 h was created and statistically analyzed (Figure 6(d)). The results showed that the enhancement induced by NPs (AUC = 46.46 ± 5.92) was significantly stronger than the enhancement induced by the MPs (AUC = 8.24 ± 6.45, *P* = 0.001).

In addition, the images of liver and kidney were also analyzed before and after injection of NPs. The results showed that there was no significant gray contrast enhancement in liver and spleen after injection of NPs (data not shown).

### 3.5. In Vivo Optical Imaging

**Figure 7:**
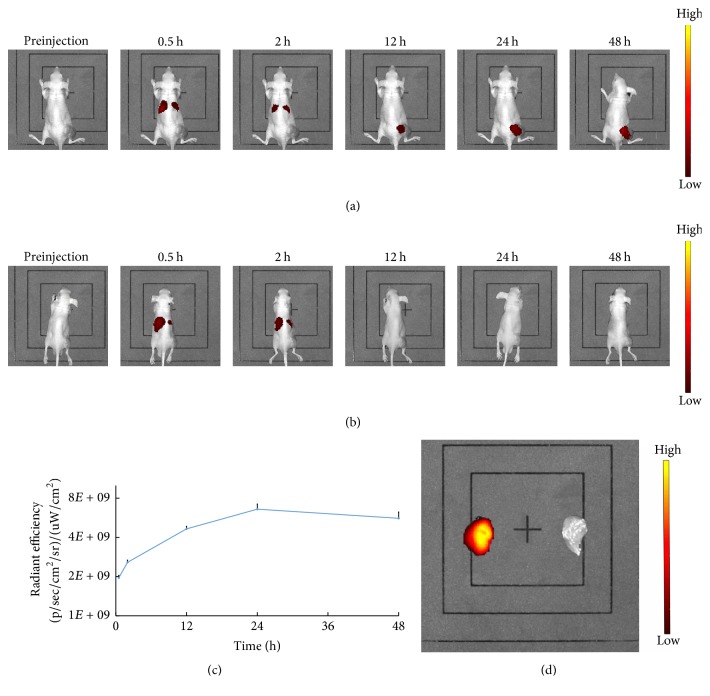
In vivo DiR fluorescence imaging results for NPs (a) group and MPs (b) group at preinjection, 0.5 h, 2 h, 12 h, 24 h, and 48 h. (c) Fluorescence intensity-time curve at tumor site after injection with NPs. (d) Comparison of DiR fluorescence in isolated tumor tissues of NP group (left) and MP group (right) at 48 h after intravenous injection.

We conducted small animals living optical imaging to further confirm that the NPs could gather in the tumor tissue. In NP group (Figure 7(a)), the red fluorescence was obviously distributed in the livers and spleens 0.5 h and 2 h after injection. 12 h after injection, significant fluorescence signals were detected at tumor site and they were still strong 48 h after injection. In the MP group, no fluorescence signals were detected in the tumor tissue (Figure 7(b)). We performed a region of interest (ROI) in the tumor tissue to analyze fluorescence enhancement which accessed the DiR uptake in each specimen (Figure 7(c)). Fluorescence signal intensity increased to the peak 24 h after injection. It slowly faded and only reduced 15% 48 h after injection. For fluorescence imaging of isolated tumors, the nude mice were sacrificed 48 h after intravenous injection. There was obvious fluorescence emission of isolated tumor tissues in NP group (left), while no fluorescence appeared in MP group (right) (Figure 7(d)).

### 3.6. Histology

**Figure 8:**
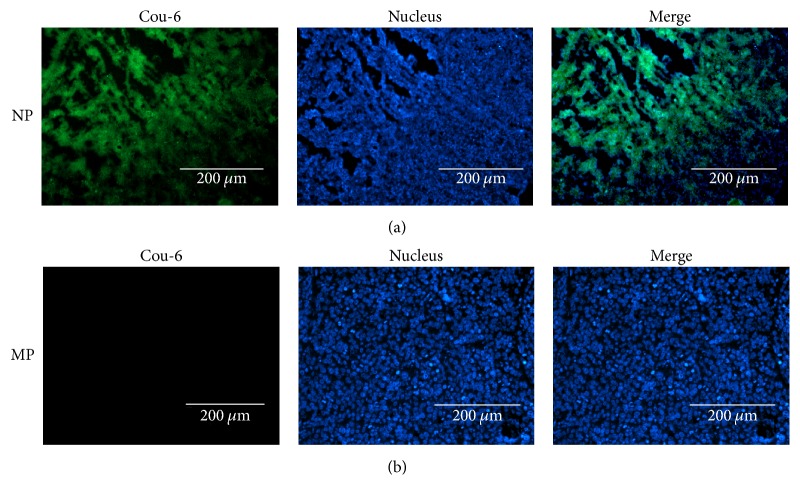
Histologic fluoroscopy images of frozen sections after nuclear labeling. Many coumarin-6-labeled NPs (green) were observed in the tumor intercellular space (a); coumarin-6-labeled MPs were hard to detect in the tumor intercellular space (b). Blue represents nuclear staining.

To further confirm the microscopic localization of NPs, the nude mice were sacrificed and frozen sections of the tumors were examined by fluorescence microscope. Tumor tissues of the nucleus were dyed blue by DAPI. NPs with green fluorescence appeared in areas beyond the nucleus in tumor tissue, but MPs with green fluorescence were not in tumor tissues (Figure 8).

## 4. Discussion

The purpose of this study is to use PFOB packaged PLGA-PEG-COOH and PLGA-COOH to prepare nanoscale UCA, which can generate contrast enhancement in vitro and in vivo. Regardless of the changes of the ratio of PLGA-PEG-COOH/PLGA-COOH, it is possible for a nanoparticle to maintain a complete shell structure by using such a mixture of membrane materials to package PFOB [18]. When the dose of PLGA-PEG was adjustable, NPs could carry enough PEG to avoid RES, so plasma half-life of NPs could be extended, and there was enough time for NPs to take advantage of EPR effect to accumulate in the tumor [19, 20]. The combination between the end of PLGA-PEG containing active carboxyl group and the end of amino target ligand let NPs have the potential for active targeting [21, 22].

PEG molecular weight of 2000 or more can avoid the RES [9]. A PEG-3400 (25 nm) spacer was used in previous studies [22]. The PEG chain density is also important in achieving improved stealth. Both high and low surface coverage of PEG chains could not avoid RES [9]. In this experiment, the density of PEG is 2%, just within threshold values (between 2 and 5 wt %) for optimal protein resistance [23].

In the preparation of nanoscale UCA, particle size and size distribution are important parameters that determine the fate of UCA in vivo studies. To get through the tumor's endothelial pore (typically between 380 and 780 nm) and escape from the RES trapping effect (i.e., NPs whose diameters were bigger than 300 nm gradually start to be trapped significantly), the optimal diameter of NPs for clinical employment should be less than 300 nm [24]. In the process of preparation of nanoparticles, the shell thickness could influence the echogenicity of contrast agents. The T/R (the thickness-to-radius ratio) was used to evaluate the shell thickness of UCA. When PFOB was packaged by PLGA at maximum, the T/R was the largest, the shell of UCA was the thinnest, and the compressibility of UCA was best and it had the highest acoustic signal [14]. However, the T/R of UCA was only related to the proportion of PLGA and PFOB in the formulation. When PLGA was 100 mg and PFOB was 60 *μ*l, the T/R was minimal. Therefore, when PFOB was >60 *μ*l, free PFOB droplets appeared [17].

Ultrasound contrast agents have the ability to enhance echogenicity. Higher concentration could produce more echo reflection and stronger echo signal. The intensity of echo reflection was directly proportional to the concentration of the particles [25]. According to Rayleigh scattering, when the particle diameter was much smaller than the wavelength, the backscatter intensity produced by the particles was proportional to the incident wave frequency to the 4th power. The higher frequency was, the greater backscattering and the stronger ultrasound intensity were seen [26].

We separately studied the effect of the MI on echo reflection. The MI is a measure of the power of an ultrasound beam. The higher the MI was valued, the greater the energy of ultrasonic emission was and the greater echo reflection results were [27]. Echogenicity was brighter at a higher MI value than at a lower MI value. Our experiment showed that NPs were stable and produced sustained ultrasonic contrast enhancement at a high MI. While microbubbles produced ultrasonic contrast enhancement when MI < 0.5 and when MI > 0.5 for transient cavitation, the microbubbles burst and produced instant and violent increases in ultrasonic reflection [28].

The ability of NPs to reflect ultrasound was almost the same as that of MPs but their time of contrast enhancement was longer than that of MPs in vivo due to the small size of NPs. The time of the contrast enhancement process was longer than 24 h, similar to Rapoport's research [29]. The time dependence of gray enhancement was based on the vascular permeability [30]. Tumor blood vessels with high permeability allowed nanoscale particles to permeate the tumor vasculature and remain in the tumor tissue. The gray contrast enhancement lasted for 24 hours after injection in tumor tissue which suggested that more NPs passed through the endothelial gaps and retained there with time going.

Target, distribution, and metabolism of contrast agents in vivo can be observed by small animals living optical imaging [31]. The tendency of the accumulated fluorescence at tumor site consistent with the tumor ultrasound imaging suggested that the NPs remained at the tumor tissue. 48 h after injection, the higher intensity of fluorescence was shown in the tumor since fluorescence agent DiR was not easy to quench in vivo [32]. Fluorescence signals were observed in livers and spleen in two groups due to reticuloendothelial system (RES) uptake. In the control group, the MPs were quickly cleared by RES, so no fluorescent signal was found in tumor tissues.

Histofluorescence imaging revealed the location of the NPs after intravenous injection. The tumors are heterogeneous. Endothelial monolayers and the cells lining of the tumor vessel are defective. This presented intercellular openings, transcellular holes, and endothelial fenestrae. The functional pore size of different tumors varied ranging from 200 nm to 2000 nm [4]. In the present research, NPs labeled Cou-6 accumulated in the tumor after penetrating through endothelial gaps, and we could observe green fluorescence in the images. However, in MP group, no green fluorescence was observed in the tumor, which meant that MPs labeled Cou-6 could not gather in the tumor by EPR. The ultrasonic imaging performance could be explained by these phenomena, where NPs penetrated tumor vessels and accumulated in the tumor at the later stage of ultrasound contrast-enhanced imaging. Thus, the time of the contrast enhancement would be much longer than that of using the MPs, which could not penetrate tumor vessels.

## 5. Conclusions

PFOB, with good echo enhancement ability, is the candidate of nanoultrasonic contrast agents. But PFOB is soluble neither in water nor in oil and it cannot be injected directly into the body, so usually it needs to be wrapped in a shell. The membrane materials that make up the shell should be able to completely package PFOB within the nanometer range, and the generated NPs have a good echo enhancement in vitro. They also enable NPs to escape the removal of RES in vivo and image after gathering in tumor tissues.

In this work, we used membrane materials, PLGA-COOH and PLGA-PEG-COOH packaged PFOB, to produce nanometer UCA. Nanoparticles were all shell-core structures. We evaluated their echogenic ability in vitro and in vivo. In vivo fluorescence imaging and frozen section further confirmed that the NPs could accumulate in tumor tissues. Their characteristics suggested that NPs may be applicable to ultrasonic molecular imaging and targeting therapy/drug/gene delivery to tumor.
